# Concerted Catalysis by Nanocellulose and Proline in Organocatalytic Michael Additions

**DOI:** 10.3390/molecules24071231

**Published:** 2019-03-29

**Authors:** Naliharifetra Jessica Ranaivoarimanana, Kyohei Kanomata, Takuya Kitaoka

**Affiliations:** Department of Agro-Environmental Sciences, Graduate School of Bioresource and Bioenvironmental Sciences, Kyushu University, 744 Motooka, Nishi-ku, Fukuoka 819-0395, Japan; jessi@agr.kyushu-u.ac.jp (N.J.R.); kanomata@agr.kyushu-u.ac.jp (K.K.)

**Keywords:** nanocellulose, TEMPO-oxidized cellulose nanofiber, Michael addition, nitroalkene, organocatalysis, proline

## Abstract

Cellulose nanofibers (CNFs) have recently attracted much attention as catalysts in various reactions. Organocatalysts have emerged as sustainable alternatives to metal-based catalysts in green organic synthesis, with concerted systems containing CNFs that are expected to provide next-generation catalysis. Herein, for the first time, we report that a representative organocatalyst comprising an unexpected combination of 2,2,6,6-tetramethylpiperidine 1-oxyl (TEMPO)-oxidized CNFs and proline shows significantly enhanced catalytic activity in an asymmetric Michael addition.

## 1. Introduction

In the last two decades, cellulose nanofibers (CNFs) have emerged as a promising state-of-the-art nanomaterial with a wide range of applications [1]. Cellulose, which is a homopolymer of D-glucose that is connected by a β-1,4 linkage, is the most abundant, carbon-neutral, and renewable natural biomass on Earth. The regular assembly of dozens of molecular cellulose chains in one direction forms highly crystalline CNFs. CNFs are mainly derived from woody secondary cell walls and they possess an uncommon combination of features, such as high strength, high transparency, a large specific surface area, a well-defined nanoarchitecture, and a highly functionalized and chemically modifiable crystalline surface [2,3]. Taking advantage of these physicochemical properties, CNFs have been widely explored in polymer nanocomposite reinforcement [4] as high performance gas-separation films and purification membranes [5,6] and in the confection of electroactive paper [7,8].

Among the reported CNFs, 2,2,6,6-tetramethylpiperidine 1-oxyl (TEMPO)-oxidized cellulose nanofibers (TOCNs) are fascinating, owing to their simple and environmentally benign preparation method and unique nanoarchitecture [9]. In TOCNs, carboxylates are introduced onto native cellulose crystals at regular intervals and with high density, resulting in a core–shell structure, in which a glucose/glucuronic acid alternating copolymer covers native crystalline cellulose bundles. Furthermore, TEMPO oxidation of wood pulp affords completely individualized cellulose microfibrils, whose widths are the narrowest within the reported CNFs (3–4 nm). Therefore, the TOCN-derived materials have superior physical properties in gas-barrier films [10], free-standing aerogels [11], and nanocomposites [12].

While the material-oriented applications of CNFs have been extensively studied, another promising application of this new nanomaterial in catalytic molecular transformations has emerged [13,14,15]. The readily functionalizable nature of cellulose makes CNFs, especially TOCNs, a matrix of choice in the immobilization of metal nanoparticles and cations as catalysts [16]. We have recently reported the novel ability of TOCNs to accelerate catalytic reactions, in which TOCNs in protonated form (TOCN-H) act as a promising solid acid catalyst for the hydrolysis of acetals in their protonated form [17]. The surface carboxylic acids on TOCN-H behave as heterogeneous acid catalysts with activity that outperforms the corresponding homogeneous catalysts, such as acetic acid. Furthermore, we reported the ability of TOCNs, in their sodium form (TOCN-Na), to improve the efficiency of proline-catalyzed aldol reactions [18]. This finding provided a new method for enhancing less-efficient proline, which is a representative organocatalyst that has attracted considerable attention, owing to its environmentally benign nature, low cost, and wide availability [19]. Adding an external catalyst to TOCNs is an attractive method, owing to its potential applicability to a wide range of catalytic reactions. However, the scope of this methodology has yet to be explored.

The Michael reaction is an important carbon–carbon/heteroatom bond forming reaction in organic synthesis [20,21,22,23,24,25]. The Michael addition of ketones to nitroalkenes affords γ-nitroketones, which are essential building blocks in the preparation of various physiologically active compounds and pharmaceuticals [24,26,27]. Although proline is a promising catalyst in terms of its cost and availability for the synthesis of such compounds, high catalyst loading is required and the enantioselectivity is poor [28,29]. Much effort has been concentrated on remedying these issues by designing more efficient catalysts that are derived from the proline structure [21,30,31], or by pairing proline with small organic molecules to form significantly more active complexes [32,33]. Methods using ionic liquids as solvents [34,35] and covalent anchoring to polymers, such as polyethylene glycol or polystyrene, to allow catalyst recovery, have also been investigated [36,37]. However, these strategies require additional catalyst preparation steps, resulting in additional costs that hamper their industrial application.

Herein, we report a new approach to enhancing the proline-catalyzed Michael reaction that takes advantage of CNFs. Adding TOCN-Na to the reaction medium greatly reduced the required proline catalyst loading and markedly enhanced the catalytic performance, rendering the process more economical, practical, and sustainable.

## 2. Results and Discussion

### 2.1. Effects of Cellulose Nanofibers on Proline-Catalyzed Michael Additions

The investigation into TOCN/proline-concerted catalysis of the Michael addition started with the reaction of cyclohexanone (**1a**) with *trans*-β-nitrostyrene (**2a**), which has been widely employed as a benchmark reaction to assess the related catalytic systems [38,39] (Table 1). The reaction with proline alone resulted in a low yield and poor enantioselectivity (entry 1). In contrast, adding the TOCN sodium salt (TOCN-Na) to the reaction medium significantly enhanced the yield under the same reaction conditions (entry 2). Notably, the diastereoselectivity and the enantioselectivity were somewhat improved in the presence of TOCNs, which is in contrast to our previous TOCN-assisted aldol reaction, resulting in poor stereoselectivity [18]. In this TOCN/proline-concerted system, an aqueous TOCN suspension was employed, replacing the water with MeOH by repetitive centrifugation before use. MeOH is known to improve the enantioselectivity of the proline-catalyzed Michael additions, although these reactions require several days to reach completion [29] (Table S1). Previous theoretical studies have shown that the explicit participation of MeOH molecules in the transition state influences the preferred approach between the enamine, which formed from proline and cyclohexanone, and nitrostyrene as the electrophile, leading to higher enantioselectivity in the formation of **3aa** [40]. Accordingly, dissolving the proline in a small portion of MeOH prior to its addition to *N*,*N*-dimethylformamide (DMF) further increased both the yield and enantioselectivity (entry 3). No additional effects were recorded while using the same amount of MeOH in the reaction without TOCNs (56% yield, *syn*:*anti* = 96:4, 34% *ee* for *syn* in 24 h). Using TOCNs alone without proline did not promote the reaction, clearly suggesting that the cooperation of catalytically inactive TOCNs and low-activity proline was critical in enhancing the reaction efficiency of this Michael addition (entry 4). Interestingly, freeze-dried TOCNs, which were poorly dispersed in the solvent, owing to severe aggregation [11], also achieved a high yield, but the enantioselectivity remained low (entry 5). The intrinsic chirality of TOCNs might not be involved in imparting enantioselectivity, because using (*R*)-proline provided the same type of enhancement (entry 6). A plausible factor was the numerous carboxylate groups that are present on the TOCN surface, as confirmed by using homogeneously dispersed sodium acetate with an equivalent carboxy group content to the TOCNs used, which also slightly augmented the reaction yield (entry 7). However, this was not a dominant factor. The influence of functional groups was also investigated while using freshly prepared TOCNs with low carboxy contents (entry 9), which resulted in lower yields, but similar improvements in enantioselectivity. Furthermore, the crystalline nature of TOCN would be essential in the present catalytic system, with amorphous carboxymethylcellulose failing to promote the reaction at all (entry 8), despite both of the cellulose derivatives containing plentiful carboxy groups in their structures. Consequently, TOCN-Na containing 1.61 mmol/g of –COONa groups (entry 3) gave the best compromise in the yield/enantioselectivity enhancements, and it was used in subsequent experiments.

### 2.2. Optimization of Reaction Conditions

Different catalyst amounts and reaction temperatures were screened to identify the optimum conditions (Table 2). A lower amount of (*S*)-proline remained effective when combined with TOCNs (Table 2, entry 1), while higher catalyst loading led to a decrease in diastereoselectivity (entry 2). The optimum loading of (*S*)-proline was 7.5 mol%, relative to nitroalkene **2a** (Table 1, entry 3). The yield and diastereoselectivity increased with an increasing temperature for reactions with and without TOCNs (Table 2, entries 3 and 4). The best result was obtained at 40 °C for 11 h in the presence of TOCNs (entry 4). With respect to green chemistry and sustainability, further reactions were conducted at room temperature, because the (*S*)-proline/TOCNs system efficiency at this temperature was close to that at 40 °C. Testing different nitrostyrene/TOCNs ratios (entries 5–9) showed that the optimum weight ratio was 1:1.34 (entry 7). Indeed, increasing the TOCN quantity from 25 mg to 100 mg (entries 5–7) improved the catalytic efficiency from modest to high, while further increasing the amount of fibers gradually impeded the reaction. This might be attributed to reaction mixture thickening that causes crowding in the reaction medium (entries 8 and 9).

### 2.3. Substrate Scope

With optimum conditions in hand, the substrate scope of the present catalyst system was investigated (Table 3). TOCNs clearly enhanced the reaction efficiency and the stereoselectivity of proline-catalyzed Michael additions of various substrates. Both electron-donating and electron-withdrawing groups on the nitrostyrene phenyl ring were compatible with the catalytic system, affording moderate to high yields and excellent diastereoselectivities in the presence of TOCNs (entries 1 and 2). In both cases the enantioselectivity was increased. Naphthyl-substituted nitroalkene, which has inherently high reactivity, as exemplified by a high yield without TOCNs, also benefited from the effect of TOCNs, which afforded increased enantioselectivity (entry 3). The generality of ketones, as the nucleophile activated by proline, was also studied in the reaction with *trans*-β-nitrostyrene. A significant increase in yield was observed for 4-oxothiane, which is a cyclic ketone bearing a heteroatom (entry 4). Unfortunately, the reaction yield with cyclopentanone was diminished in the presence of TOCNs, despite markedly increased enantioselectivity (entry 5). Acetone, the simplest ketone without a cyclic structure, was also applicable to the present catalytic system (entry 6).

## 3. Materials and Methods

### 3.1. General

Nippon Paper Industries Co., Ltd. (Tokyo, Japan) kindly supplied TEMPO-oxidized cellulose nanofibers (TOCNs, carboxylate content: 1.61 mmol/g), and they were used in reactions, except for Table 1, entry 9. TOCNs with a low carboxylate content used in Table 1, entry 8, were prepared from cellulose nanofibers purchased from Sugino Machine Limited (BiNFi-s AFo-10002, Uozu, Japan) according to a literature method [17,41]. Transmission electronic microscopy (TEM) with a JEM-2100HCKM microscope (JEOL, Tokyo, Japan) at the Ultramicroscopy Research Center, Kyushu University and powder X-ray diffraction (XRD) using a Rigaku MultiFlex diffractometer (Rigaku Corporation., Tokyo, Japan) at the Center of Advanced Instrumental Analysis, Kyushu University (see Figures S1 and S2, respectively, in Supplementary Materials) characterized the TOCN samples. The substrates **2c** and **2d** were synthesized according to a previously-reported method (detailed in the Supplementary Materials) [42]. Other reagents were purchased from Sigma-Aldrich Co. LLC. (Tokyo, Japan), FUJIFILM Wako Pure Chemical Industries, Ltd. (Osaka, Japan), and Tokyo Chemical Industry Co., Ltd. (Tokyo, Japan), and they were used without further purification. Thin-layer chromatography (TLC) analysis was performed on glass-backed plates precoated with silica gel (silica gel 60 GF254, 0.25 mm Merck, Tokyo, Japan). Column chromatography was performed while using an automated flash chromatography system (Smart Flash EPCLC-AI-580S, Yamazen, Osaka, Japan). The ^1^H- and ^13^C-NMR spectra were recorded on a JNM-ECZ400 spectrometer (JEOL, Tokyo, Japan) at the Center of Advanced Instrumental Analysis, Kyushu University. Chemical shifts in the ^1^H-NMR spectra are reported in parts per million relative to the peak of tetramethylsilane (TMS, δ 0.00 ppm), used as an internal standard. The ^1^H-NMR data are reported, as follows: chemical shift, multiplicity (s = singlet, d = doublet, t = triplet, q = quartet, m = multiplet), coupling constants (Hz), and integration. ^13^C-NMR spectra were recorded with complete proton decoupling, and chemical shifts are reported in parts per million relative to the solvent resonance (CDCl_3_, δ 77.0 ppm), used as an internal standard.

### 3.2. Preparation of TOCNs with Low Carboxylate Content

Wet-state cellulose nanofibers (3.0 g dry weight of cellulose) were suspended in deionized water (300 mL) containing TEMPO (48 mg) and NaBr (300 mg). TEMPO-mediated oxidation was started by adding sodium hypochlorite (2.0 mmol/g of cellulose) to the suspension. The mixture was stirred at room temperature for 30 min, while the suspension was maintained at pH 10 while using a pH titrator (GT-200 Automatic Titrator, Mitsubishi Chemical Analytech, Yamato, Japan) that was loaded with NaOH (0.5 M). The oxidation was quenched with ethanol (10 mL) and the suspension was washed thoroughly with deionized water by successive centrifugation at 22,540× *g* for 10 min (TOMMY Suprema 21 High-Speed Refrigerated Centrifuge, TOMMY Seiko, Tokyo, Japan). Nanofibrillation was conducted by aqueous counter collision with a high-pressure water jet system at 245 MPa (0.1-mm diameter dual-nozzle chamber; Star Burst Labo, Sugino Machine Limited, Uozu, Japan) [43]. The obtained mixture was then centrifuged to separate the cellulose nanofiber as supernatant (0.6 wt%) from the unfibrillized fibers (10 min, 22,540× *g*). Electrical conductivity titration confirmed the carboxylate content of the TOCNs [41].

### 3.3. Water Removal Process from Nanocellulose Samples

Each nanocellulose was subjected to solvent exchange to remove water prior to use, because water inhibited the reaction, except for that in entry 4 in Table 1, which was then freeze-dried. To obtain freeze-dried samples, TOCN water suspension (10 mL, 1.04 wt%) was mixed with tertiary butanol (30 mL), frozen in liquid nitrogen, and dried under vacuum (Scanvac CoolSafe LaboGene, Sakuma Seisakusho, Co. Ltd., Tokyo, Japan). Washing the TOCN water suspension (10 mL, 1.04 wt%) with MeOH (30 mL) by centrifugation (9200× *g*, 5 min) five times was undertaken to perform the solvent exchange process.

### 3.4. Representative Procedure for the Proline-Catalyzed Michael Addition of Cyclohexanone to trans-β-Nitrostyrene in the Presence of TOCNs

Cyclohexanone (**1a**) (4.0 mL, excess) and *trans*-β-nitrostyrene (**2a**) (74.6 mg, 0.50 mmol) were added to the reaction medium, followed by a solution of (*S*)-proline (4.4 mg, 7.5 mol% with respect to **2a**) in MeOH (2.0 mL). The resulting mixture was stirred at room temperature and TLC monitored progress. Upon completion, the reaction was quenched by adding aq. NH_4_Cl, extracted with dichloromethane (30 mL × 3), and dried over Na_2_SO_4_. Purification of the concentrated organic layer by column chromatography (hexane and ethyl acetate as eluents) rendered product **3aa** as a white solid (108.8 mg, 88%). The enantiomeric and diastereomeric ratios of the reactions were measured by supercritical fluid chromatography (SFC) while using a chiral stationary phase (ACQUITY UPC2, Waters, Tokyo, Japan). The spectroscopic data of each product were in agreement with previously reported data [39,44].

#### Spectroscopic Data

*(S)-2-((R)-2-Nitro-1-phenylethyl)cyclohexanone* (**3aa**) [44]. White solid (108.8 mg, 88%); ^1^H-NMR (400 MHz, CDCl_3_) *syn*-**3aa**: δ 7.34–7.28 (m, 3H), 7.18–7.15 (m, 2H), 4.94 (dd, *J* = 12.4, 4.4 Hz, 1H), 4.64 (dd, *J* = 12.4, 10.0 Hz, 1H), 3.76 (dd, *J* = 10.0, 4.8 Hz, 1H), 2.73–2.66 (m, 1H), 2.51–2.35 (m, 2H), 2.12–2.04 (m, 1H), 1.82–1.51 (m, 4H), 1.29–1.19 (m, 1H); detectable peaks of *anti*-**3aa**: δ 4.89–4.81 (m, 0.1H), 4.04–3.99 (m, 0.05H), 2.76–2.75 (m, overlapped with *syn*-**3aa**, 0.04H), 2.30–2.24 (m, overlapped with *syn*-**3aa**, 0.02H), 1.92–1.84 (m, 0.04H), 1.43–1.34 (m, 0.05H); ^13^C-NMR (100.5 MHz, CDCl_3_) *syn*-**3aa**: δ 211.9, 137.7, 128.8, 128.1, 127.6, 78.8, 52.4, 43.8, 42.6, 33.1, 28.4, 24.9; detectable peaks of *anti*-**3aa**: δ 210.4, 138.3, 128.6, 128.2, 127.4, 76.4, 53.7, 42.9, 42.2, 29.8, 27.3; SFC: Daicel Chiralpak IC-3 (λ = 210 nm, CO_2_/2-propanol, 93:7, 1.0 mL/min, 30 °C), t_r_ (*major*) = 3.56 min, t_r_ (*minor*) = 4.70 min.

*(S)-2-((R)-1-(4-Methoxyphenyl)-2-nitroethyl)cyclohexanone* (**3ab**) [44]. Yellow solid (94.6 mg, 68%); ^1^H-NMR (400 MHz, CDCl_3_) *syn*-**3ab**: δ 7.08 (d, *J* = 8.4 Hz, 2H), 6.85 (d, *J* = 8.8 Hz, 2H), 4.91 (dd, *J* = 12.4, 4.8 Hz, 1H), 4.58 (dd, *J* = 12.4, 10.0 Hz, 1H), 3.78 (s, 3H), 3.74–3.68 (m, 1H), 2.68–2.61 (m, 1H), 2.50–2.34 (m, 2H), 2.11–2.04 (m, 1H), 1.82–1.52 (m, 4H), 1.28–1.18 (m, 1H); detectable peaks of *anti*-**3ab**: δ 7.20–7.16 (m, 0.09H), 4.87–4.71 (m, 0.13H), 3.96–3.88 (m, 0.06H); ^13^C-NMR (100.5 MHz, CDCl_3_) *syn*-**3ab**: δ 212.0, 158.8, 129.4, 129.0, 114.1, 79.0, 55.1, 52.5, 43.1, 42.6, 33.0, 28.4, 24.9; detectable peaks of *anti*-**3ab**: δ 210.6, 158.7, 129.3, 113.9, 76.9, 53.7, 42.4, 42.2, 30.0, 27.2; SFC: Daicel Chiralpak AD-3, (λ = 220 nm, CO_2_/methanol, 95:5, 1.0 mL/min, 30 °C), t_r_ (*minor*) = 5.90 min, t_r_ (*major*) = 8.61 min.

*(S)-2-((R)-1-(4-Bromophenyl)-2-nitroethyl)cyclohexanone* (**3ac**) [44]. White solid (135.7 mg, 83%); ^1^H-NMR (400 MHz, CDCl_3_) *syn*-**3ac**: δ 7.48–7.43 (m, 2H), 7.20–7.04 (m, 2H), 4.93 (dd, *J* = 12.8, 4.6 Hz, 1H), 4.60 (dd, *J* = 12.8, 10.1 Hz, 1H), 3.75 (td, *J* = 9.8, 4.4 Hz, 1H), 2.68–2.62 (m, 1H), 2.50–2.45 (m, 1H), 2.42–2.33 (m, 1H), 2.12–2.06 (m, 1H), 1.83–1.55 (m, 4H), 1.23 (qd, *J* = 12.4, 3.5 Hz, 1H); detectable peaks of *anti*-**3ac**: δ 7.44–7.43 (m, 0.26H), 7.16–7.14 (m, 0.21H), 4.89–4.78 (m, 0.21H), 3.94–3.89 (m, 0.12H), 2.75–2.70 (m, overlapped with *syn*-**3ac**, 0.11H), 1.94–1.87 (m, 0.13H), 1.42–1.31 (m, 0.11H); ^13^C-NMR (100.5 MHz, CDCl_3_) *syn*-**3ac**: δ 211.4, 136.8, 131.9, 129.8, 121.5, 78.4, 52.1, 43.3, 42.6, 33.0, 28.3, 24.9; detectable peaks of *anti*-**3ac**: δ 210.2, 137.3, 131.7, 130.1, 76.4, 53.4, 42.2, 30.0, 27.2; SFC: Daicel Chiralpak IC-3, (λ = 220 nm, CO_2_/2-propanol, 95:5, 1.0 mL/min, 30 °C), t_r_ (*major*) = 6.22 min, t_r_ (*minor*) = 9.62 min.

*(S)-2-((R)-1-Naphthyl-2-nitroethyl)cyclohexanone* (**3ad**) [44]. Off-white solid (105.1 mg, 71%); ^1^H-NMR (400 MHz, CDCl_3_) *syn*-**3ad**: δ 7.82–7.77 (m, 3H), 7.67 (d, *J* = 26.5 Hz, 1H), 7.49–7.44 (m, 2H), 7.30–7.25 (m, 1H), 5.03 (dd, *J* = 12.6, 4.3 Hz, 1H), 4.72 (dd, *J* = 12.8, 10.1 Hz, 1H), 3.95 (td, *J* = 10.1, 4.1 Hz, 1H), 2.76 (td, *J* = 11.1, 4.6 Hz, 1H), 2.49–2.32 (m, 2H), 2.05–2.00 (m, 1H), 1.71–1.49 (m, 4H), 1.28–1.19 (m, 1H); detectable peaks of *anti*-**3ad**: δ 7.69 (s, 0.12H), 7.39–7.36 (m, 0.11H), 4.96–4.93 (m, 0.13H), 4.20–4.15 (m, 0.08H), 2.24 (m, 0.07H), 1.85–1.76 (m, 0.08H), 1.42–1.29 (m, overlapped with *syn*-**3ad**, 0.04H); ^13^C-NMR (100.5 MHz, CDCl_3_) *syn*-**3ad**: δ 211.8, 135.0, 133.2, 132.7, 128.7, 127.7, 127.5, 126.3, 126.0, 125.1, 78.7, 52.3, 44.0, 42.6, 33.2, 28.4, 24.9; detectable peaks of *anti*-**3ad**: δ 210.4, 135.8, 133.1, 132.5, 128.4, 127.5, 126.9, 126.4, 126.2, 126.0, 76.4, 53.7, 42.9, 42.2, 29.8, 27.2; SFC: Daicel Chiralpak OD-3, (λ = 210 nm, CO_2_/2-propanol, 82:18, 1.0 mL/min, 30 °C), t_r_ (*major*) = 1.34 min, t_r_ (*minor*) = 1.73 min.

*(S)-3-((R)-2-Nitro-1-phenylethyl)tetrahydro-4H-thiopyran-4-one* (**3ba**) [39]. White solid (100.8 mg, 76%); ^1^H-NMR (400 MHz, CDCl_3_) *syn*-**3ba**: δ 7.36–7.17 (m, 5H), 4.73 (dd, *J* = 12.8, 4.6 Hz, 1H), 4.61 (dd, *J* = 12.8, 9.6 Hz, 1H), 3.96 (td, *J* = 10.3, 4.7 Hz, 1H), 3.06–2.93 (m, 3H), 2.87–2.77 (m, 2H), 2.62–2.41 (m, 2H); detectable peaks of *anti*-**3ba**: δ 4.90–4.81 (m, 0.02H), 4.18–4.13 (m, 0.03H), 2.94–2.92 (m, overlapped with *syn*-**3ba**, 0.06H); ^13^C-NMR (100.5 MHz, CDCl_3_) *syn*-**3ba**: δ 209.5, 136.4, 129.3, 128.3, 128.1, 78.6, 54.9, 44.5, 43.4, 35.1, 31.6; detectable peak of *anti*-**3ba**: δ 129.0; SFC: Daicel Chiralpak IC-3, (λ = 210 nm, CO_2_/methanol, 96:4, 1.0 mL/min, 30 °C), t_r_ (*major*) = 2.41 min, t_r_ (*minor*) = 2.75 min.

*(S)-2-((R)-2-Nitro-1-phenylethyl)cyclopentan-1-one* (**3ca**) [44]. Off-white solid (13.7 mg, 12%); ^1^H-NMR (400 MHz, CDCl_3_) *syn*-**3ca**: δ 7.32 (s, 3H), 7.14 (s, 2H), 5.32 (dd, *J* = 12.8, 5.5 Hz, 1H), 5.00 (d, *J* = 7.8 Hz, 1H), 4.70 (dd, *J* = 12.8, 10.1 Hz, 1H), 3.82 (dd, *J* = 11.9, 7.8 Hz, 1H), 3.71–3.65 (m, 1H), 2.36 (d, *J* = 43.9 Hz, 1H), 2.12 (d, *J* = 41.6 Hz, 1H), 1.89 (d, *J* = 49.4 Hz, 1H), 1.70 (d, *J* = 34.8 Hz, 1H), 1.47 (d, *J* = 41.6 Hz, 1H); detectable peaks of *anti*-**3ca**: δ 7.42–7.41 (m, 0.12H), 7.13–7.05 (m, 0.18H), 3.83 (td, *J* = 7.8, 4.1 Hz, 0.34H), 2.29–2.25 (m, overlapped with *syn*-**3ca**, 0.16H), 1.41–1.20 (m, 0.17H); ^13^C-NMR (100.1 MHz, CDCl_3_) *syn*-**3ca**: δ 218.5, 137.7, 128.9, 128.4, 127.9, 78.2, 50.4, 44.1, 38.6, 28.3, 20.0; detectable peaks of *anti*-**3ca**: δ 219.1, 137.3, 128.9 (overlapped with *syn*-**3ca**), 77.1, 51.4, 44.0, 39.2, 27.0, 20.5; SFC: Daicel Chiralpak IA-3, (λ = 210 nm, CO_2_/2-propanol, 95:5, 1.0 mL/min, 30 °C), t_r_ (*minor*) = 1.70 min, t_r_ (*major*) = 2.56 min.

*(R)-5-Nitro-4-phenylpentan-2-one* (**3da**) [39]. White solid (72.3 mg, 70%); ^1^H-NMR (400 MHz, CDCl_3_): δ 7.33–7.19 (m, 5H), 4.62 (ddd, *J* = 40.0, 12.3, 7.5 Hz, 2H), 4.03–3.95 (m, 1H), 2.89 (d, *J* = 7.3 Hz, 2H), 2.09 (s, 3H); ^13^C-NMR (100.5 MHz, CDCl_3_): δ 205.4, 138.8, 128.9, 127.7, 127.3, 79.3, 46.0, 38.9, 30.2; SFC: Daicel Chiralpak IC-3, (λ = 210 nm, CO_2_/2-propanol, 93:7, 1.0 mL/min, 30 °C), t_r_ (*major*) = 3.05 min, t_r_ (*minor*) = 3.50 min.

## 4. Conclusions

We have developed a novel method that substantially enhanced the proline-catalyzed Michael additions of ketones to nitroalkenes through the simple incorporation of nanocellulose, TOCNs, in the reaction medium at room temperature. We assumed that crystalline TOCNs with densely packed surface carboxylate groups played a significant role in increasing the catalytic activity, because TOCNs with low carboxylate content only gave a small yield enhancement. In addition, molecular carboxylates that originated from sodium acetate gave a modestly improved yield, while the amorphous carboxylated cellulose derivative was not effective. Furthermore, the efficiency of the catalytic system was directly related to the amount of nanocellulose used. The method was successfully applied to different ketones and nitroalkene substrates. Concerted catalysis using nanocelluloses and organocatalysts provides new research avenues for both emerging applications of nanocellulose and green sustainable chemistry.

## Figures and Tables

**Table 1:**
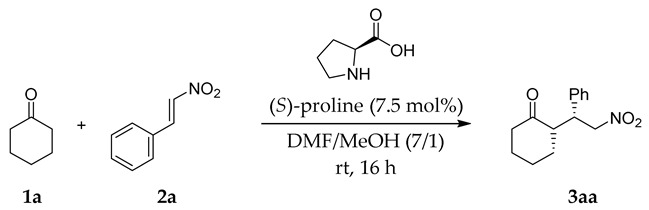
Catalytic behavior of proline combined with cellulose nanofibers and additives in the Michael addition ^a^.

Entry	Additive	Yield (%) ^b^	*syn*:*anti*^c^	*ee* for *syn* (%) ^c^
1	None	35	89:11	32
2 ^d^	TOCN	78	90:10	35
3	TOCN	88	90:10	43
4 ^e^	TOCN	Trace	-	-
5 ^f^	TOCN	81	95:5	33
6 ^g^	TOCN	86	93:7	-39
7	Sodium acetate	41	89:11	6
8 ^h^	Carboxymethylcellulose	34	90:10	27
9 ^i^	TOCN/low carboxylate	42	82:18	42

^a^ Unless otherwise noted, the reaction was performed using cyclohexanone (**1a**) (4 mL, excess), *trans*-β-nitrostyrene (**2a**) (74.6 mg, 0.50 mmol), (*S*)-proline (7.5 mol%), and 2,2,6,6-tetramethylpiperidine 1-oxyl (TEMPO)-oxidized cellulose nanofibers-Na (TOCN-Na) (100 mg dry weight) in a mixture of DMF (14 mL) and MeOH (2 mL). Aqueous medium of TOCN suspension was replaced with MeOH by repetitive centrifugation prior to reaction; ^b^ Isolated yield; ^c^ Determined by chiral stationary phase supercritical fluid chromatography (SFC) analysis; ^d^ Without MeOH; ^e^ Without (*S*)-proline; ^f^ Freeze-dried TOCN was added to the reaction; ^g^ (*R*)-Proline was used instead of (*S*)-proline; ^h^ For entries 6 and 7, additive amount adjusted to 1 eq. of –COO^−^ groups contained in 100 mg of TOCN (entry 3); ^i^ TOCN with –COO^−^ group content of 0.94 mmol/g.

**Table 2:**
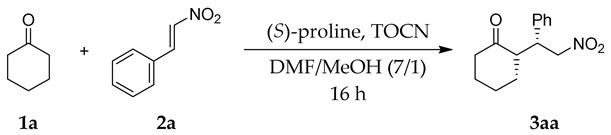
Fine-tuning of catalyst amount, reaction temperature, and cellulose nanofiber quantity ^a^.

Entry	Conditions	TOCN	Yield (%) ^b^	*syn*:*anti*^c^	*ee* for *syn* (%) ^c^
1	5 mol% catalyst, rt	−	37	87:13	29
		+	74	93:7	39
2	15 mol% catalyst, rt	−	58	69:31	26
		+	77	69:31	39
3	7.5 mol% catalyst, 0 °C	−	9	77:23	19
		+	23	74:26	39
4 ^d^	7.5 mol% catalyst, 40 °C	−	66	94:6	32
		+	91	94:6	42
5	Substrate/TOCN (mg/mg) 74.6/25	+	51	92:8	41
6	Substrate/TOCN (mg/mg) 74.6/50	+	66	94:6	43
7	Substrate/TOCN (mg/mg) 74.6/100	+	88	90:10	41
8	Substrate/TOCN (mg/mg) 74.6/150	+	63	71:29	41
9	Substrate/TOCN (mg/mg) 74.6/200	+	43	83:17	43

^a^ Unless otherwise noted, the reaction was performed using cyclohexanone (**1a**) (4 mL, excess), *trans*-β-nitrostyrene (**2a**) (74.6 mg, 0.50 mmol), (*S*)-proline (7.5 mol%), and TOCN-Na (100 mg dry weight) in a mixture of DMF (14 mL) and MeOH (2 mL). Aqueous medium of TOCN suspension was replaced with MeOH by repetitive centrifugation prior to reaction; ^b^ Isolated yield; ^c^ Determined by chiral stationary phase SFC analysis; ^d^ Stirred for 11 h.

**Table 3:**
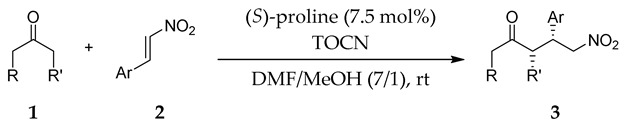
Substrate scope ^a^.

Entry	Substrates	Product	Time (h)	TOCN	Yield (%) ^b^	*syn*:*anti*	*ee* for *syn* (%) ^c^
1	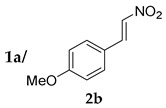	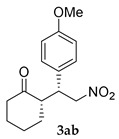	48	−	26	90:10 ^d^	31
			48	+	68	93:7 ^d^	35
2	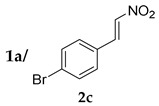	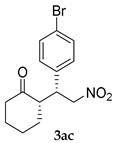	24	−	59	96:4 ^c^	28
			24	+	83	95:5 ^c^	50
3	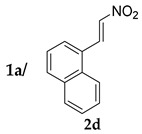	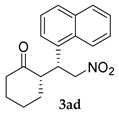	48	−	75	87:13 ^d^	10
			48	+	71	87:13 ^d^	21
4	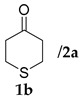	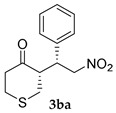	24	−	18	94:6 ^d^	24
			24	+	76	97:3 ^d^	38
5	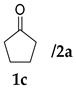	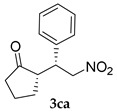	24	−	43	61:39 ^c^	29
			24	+	12	72:28 ^c^	59
6	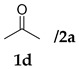	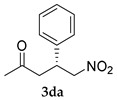	24	−	32	-	10
			24	+	70	-	23

^a^ Unless otherwise noted, the reactions were performed using an excess of ketone **1** (4 mL for all entries, except 10 eq. of **1b** in entry 4) and nitrostyrene (**2**) (0.50 mmol); ^b^ Isolated yield; ^c^ Determined by chiral stationary phase SFC analysis; ^d^ Determined by ^1^H-NMR.

## Supplementary Materials

The supplementary materials are available online.
