# Supplementary material for: Concerted Catalysis by Nanocellulose and Proline in Organocatalytic Michael Additions
Source: Molecules. 2019 Mar 29;24(7):1231. doi: 10.3390/molecules24071231 (PMC6480416; doi:10.3390/molecules24071231)
Supplement: Supplementary file 1 [file molecules-24-01231-s001.pdf]

Supplementary Materials

Concerted Catalysis by Nanocellulose and Proline in Organocatalytic Michael Additions

Naliharifetra Jessica Ranaivoarimanana, Kyohei Kanomata and Takuya Kitaoka*

Department of Agro-Environmental Sciences, Graduate School of Bioresource and Bioenvironmental Sciences, Kyushu University, 744 Motooka, Nishi-ku, Fukuoka 819-0395, Japan
*Correspondence: tkitaoka@agr.kyushu-u.ac.jp; Tel.: +81-92-802-4665

Contents

Characterization of cellulose nanofibers 2

Table S1. Solvent screening[a] 3

Substrate synthesis 4

Supercritical fluid chromatography (SFC) profiles 5

Appendix: List of reagents and chemicals 12

References 13

Characterization of cellulose nanofibers

Transmission electron microscopy (TEM) was performed using a JEM-2100HCKM microscope (JEOL, Tokyo, Japan), operated at an accelerating voltage of 200 kV, at the Ultramicroscopy Research Center Kyushu University. A TOCN dispersion (0.005% w/w, 5 μL) was mounted on a glow-discharged carbon-coated Cu grid. The excess liquid was absorbed with a filter paper after 5 min, and a negative staining reagent (1% sodium phosphotungstate, 5 μL), was dropped onto the sample. After standing for 5 min, the excess liquid was absorbed with a filter paper. Deionized water (5 μL) was added dropwise, and removed with a filter paper after 3 min. After air-drying, the coated grid was observed using the TEM apparatus in bright-field mode.

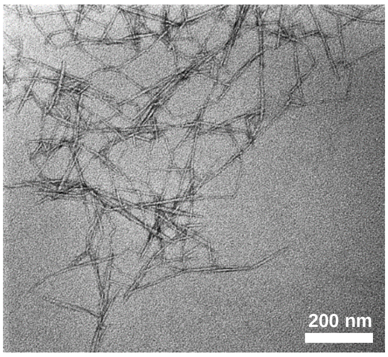

Figure S1. TEM images of TOCNs supplied by Nippon Paper Industries Co., Ltd (Tokyo, Japan).

X-Ray diffraction (XRD) patterns were recorded using a Rigaku MultiFlex diffractometer (Rigaku Corporation, Tokyo, Japan) with Ni-filtered Cu Kα radiation (λ= 0.1528 nm) at 40 kV and 20 mA. Scanning was performed at 0.5° min^{-1} with 0.05° intervals. Freeze-dried TOCN samples were pressed to make a pellet. The crystallinity index (CrI) was calculated according to the method reported by Segal and co-workers [1]:

$$\mathrm{CrI}\ (\%) = [(I_{200}–I_{am})/I_{200}] \times 100 \qquad (1)$$

where I_{200} represents the intensity at 2θ = 22.5° and I_{am} at 2θ = 18.7°. The sample crystallite dimension (D_{200}) was calculated using the Scherrer equation:

$$D_{200} = 0.9\lambda/\beta\cos\theta \qquad (2)$$

where β is the line broadening at the full width at half maximum (FWHM) after subtracting the instrumental line broadening, in radians.

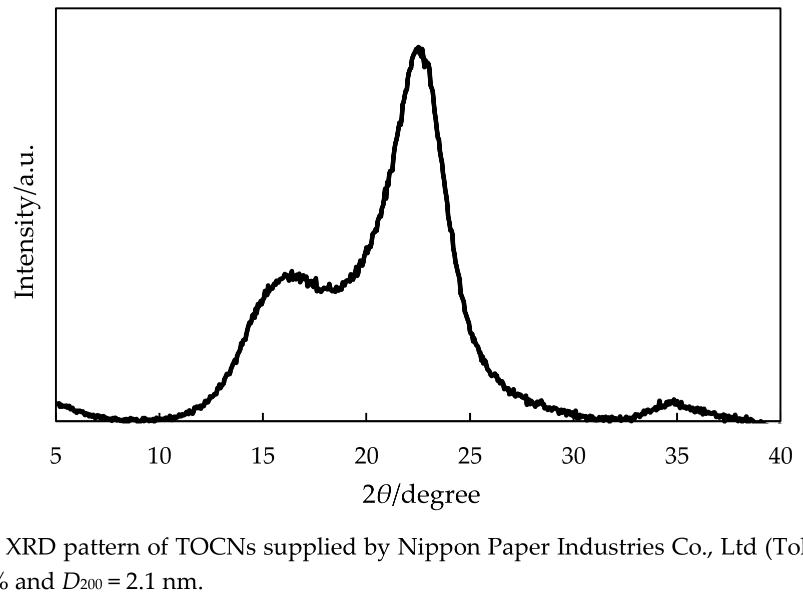

Figure S2. XRD pattern of TOCNs supplied by Nippon Paper Industries Co., Ltd (Tokyo, Japan). CrI = 57.6% and D_{200} = 2.1 nm.

The carboxylate content of the TOCNs was determined by conductometric titration method [2]. A freeze-dried sample (ca. 100 mg) was added to deionized water (55 mL) and 0.02 M NaCl (5 mL), and the mixture was vigorously stirred to prepare a well-dispersed slurry. Then, 0.1 M HCl was added to the mixture to set the pH value in the range of 2.5–3.0. A 0.05 M NaOH solution was added at the rate of 0.2 mL/min with a Fusion 100 CX07100 micro syringe pump (Chemyx Inc., Stafford, United States of America) until the pH raised up to 11. The pH and conductivity were monitored by a LAQUA F-74 (HORIBA Advanced Techno Co., Ltd, Kyoto, Japan) during titration. The carboxylate content (mmol/g) of the sample was determined from the pH and conductivity curves using the following equation:

$$\text{COONa content} = V_e C_{NaOH} / W \quad (3)$$

where V_e is the titration volume (mL) determined by the conductometric curve, C_{NaOH} is the molar concentration of NaOH and W is the amount of freeze-dried TOCN sample (g). The COONa content was determined as the average of three titrations.

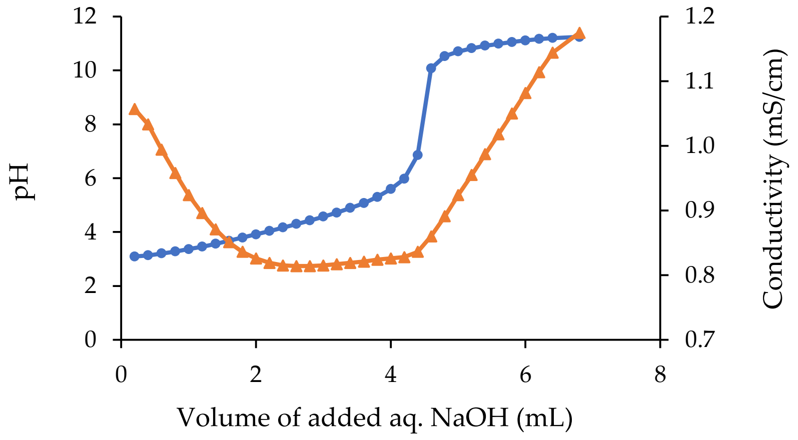

Figure S3. Representative pH (circles) and electrical conductivity (triangles) curves of an aqueous suspension of TOCNs supplied by Nippon Paper Industries Co., Ltd (Tokyo, Japan). Carboxylate content: 1.61 mmol/g of the cellulose sample.

Table S1. Solvent screening[a]

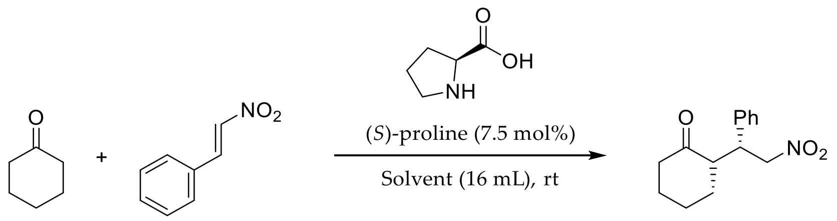

1a **2a** **3aa**

Entry	Solvent	Time (h)	TOCN	Yield (%)[b]	*syn* : *anti*[c]	*ee* for *syn* (%)[c]
1	H_2O	48	-	Trace	-	-
			+	Trace	-	-
2	MeOH	96	-	11	96:4	39
			+	42	97:3	45
3	IPA	48	-	55	96:4	33
			+	17	97:3	52

4	DCM	48	-	Trace	-	-
			+	Trace	-	-
5	MeCN	48	-	Trace	-	-
			+	Trace	-	-
6	DMSO	16	-	57	95:5	32
			+	73	95:5	29
7	DMF	16	-	35	89:11	32
			+	88	90:10	43

[a] Otherwise stated, the reaction was performed using cyclohexanone (**1a**) (4 mL, excess), *trans*-β-nitrostyrene (**2a**) (74.6 mg, 0.50 mmol), (*S*)-proline (7.5 mol%), and TOCN-Na (100 mg in dry weight) in the adequate solvent (16 mL). Aqueous medium of TOCN suspension was replaced by MeOH by repetitive centrifugation prior to the reaction; [b] Isolated yield; [c] Determined by chiral stationary phase supercritical fluid chromatography (SFC) analysis.

Substrate synthesis

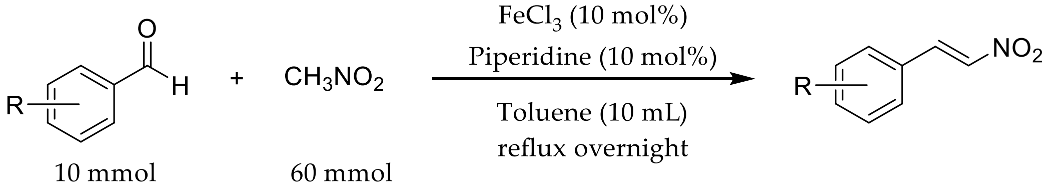

All reagents used for the substrate synthesis were purchased from FUJIFILM Wako Pure Chemical Industries, Ltd. (Osaka, Japan), Merck KGaA (Darmstadt, Germany), Sigma-Aldrich Co. LLC. (Tokyo, Japan), and Tokyo Chemical Industry Co., Ltd. (Tokyo, Japan), and used without further purification. The adequate substituted benzaldehyde (10.0 mmol), CH_3NO_2 (4 mL, 60 mmol), and piperidine (98.8 μL, 1.0 mmol) were added sequentially to an oven-dried round-bottom flask (100 mL) containing toluene (10 mL), then anhydrous $FeCl_3$ (16.2 mg, 0.10 mmol) was poured in. The mixture was refluxed under N_2 atmosphere overnight. After being cooled down to room temperature, the reaction mixture was concentrated under reduced pressure. Column chromatography (hexane and dichloromethane as eluent) of the concentrated organic layer afforded the product. The spectroscopic data of each product were in agreement with previously reported data [3]:

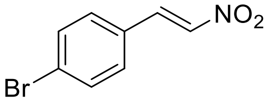

(E)-1-Bromo-4-(2-nitrovinyl)benzene (**2c**). Brown solid; ^{1}H-NMR (400 MHz, $CDCl_3$): δ_H = 7.95 (d, *J* = 13.7 Hz, 1H), 7.61−7.58 (m, 3H), 7.43−7.40 (d, *J* = 8.7 Hz, 2H); ^{13}C-NMR (100.5 MHz, $CDCl_3$): δ_C = 137.8, 137.4, 132.7, 130.4, 128.9, 126.7.

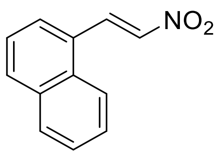

(E)-1-(2-Nitrovinyl)naphthalene (**2d**). Yellow solid; ^{1}H-NMR (400 MHz, $CDCl_3$): δ_H = 8.10 (d, *J* = 13.7 Hz, 1H), 7.94 (s, 1H), 7.87−7.82 (m, 3H), 7.65 (d, *J* = 13.7 Hz, 1H), 7.59−7.52 (m, 3H); ^{13}C-NMR (100.5 MHz, $CDCl_3$): δ_C = 139.1, 137.0, 134.8, 133.02 132.2, 129.3, 128.8, 128.3, 127.9, 127.4, 127.2, 123.2.

Supercritical fluid chromatography (SFC) profiles

Each product is described with a set of two SFC profiles. The first is of the racemic product, and the second, the asymmetric. The vertical axis is the absorbance in atomic units (AU) and the horizontal axis is the retention time in minute (min).

(S)-2-((R)-2-Nitro-1-phenylethyl)cyclohexanone (**3aa**)

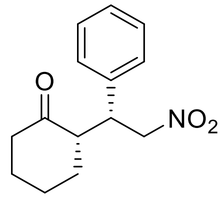

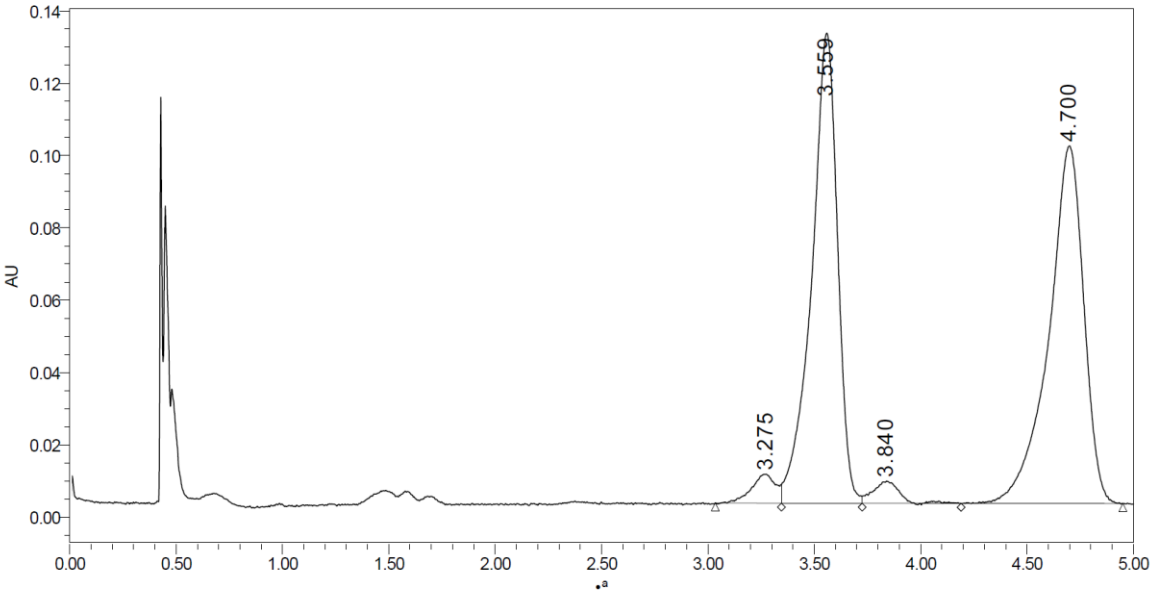

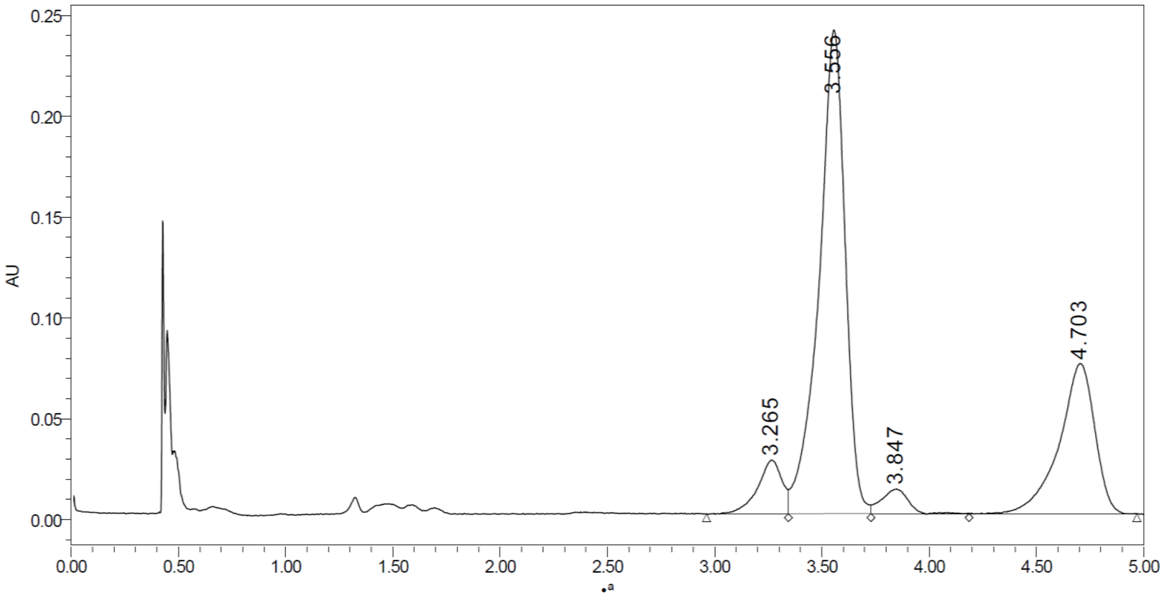

(S)-2-((R)-1-(4-Methoxyphenyl)-2-nitroethyl)cyclohexanone (**3ab**)

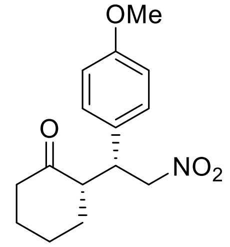

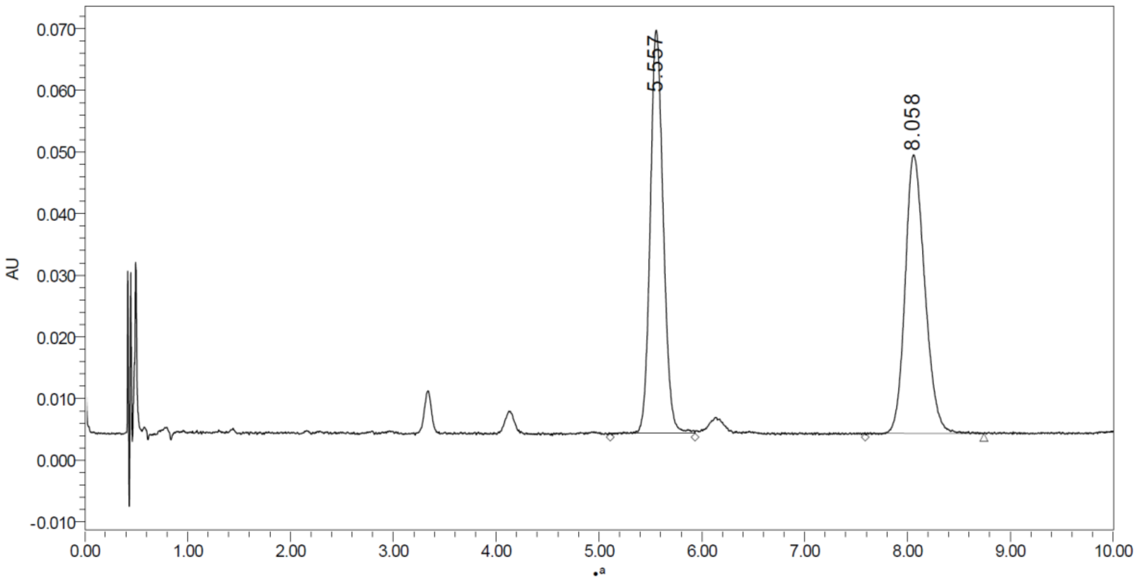

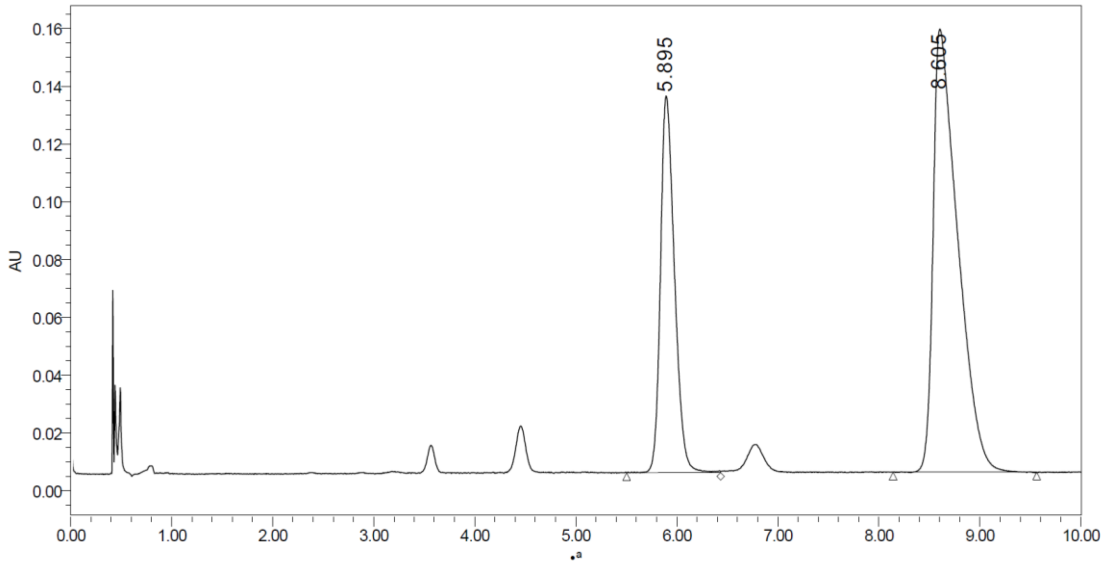

(S)-2-((R)-1-(4-Bromophenyl)-2-nitroethyl)cyclohexanone (**3ac**)

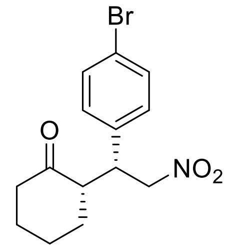

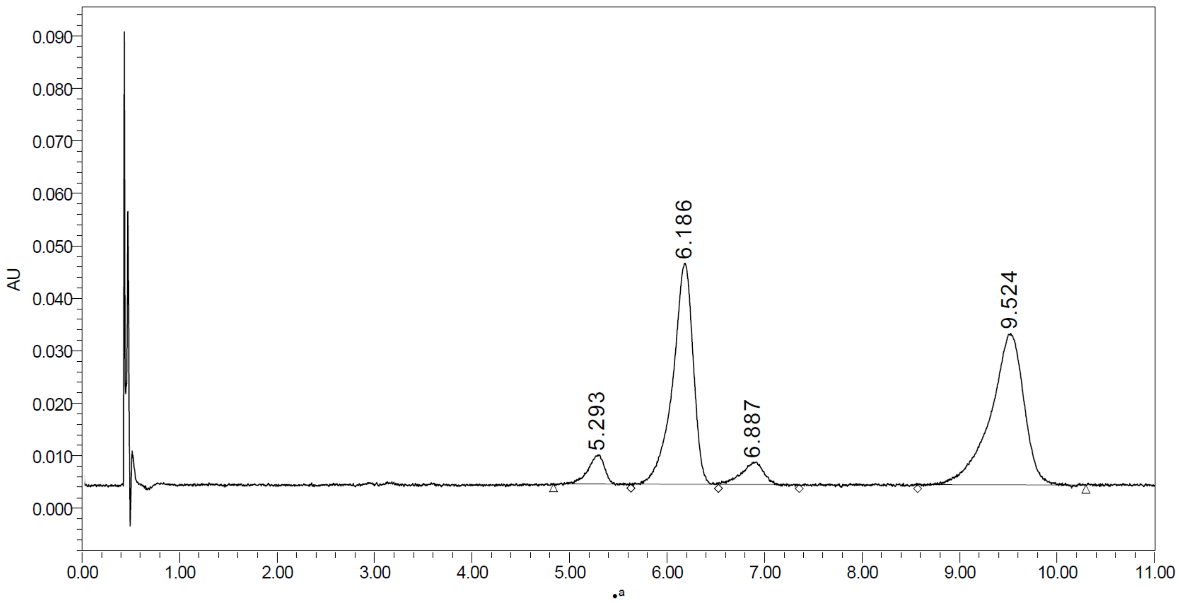

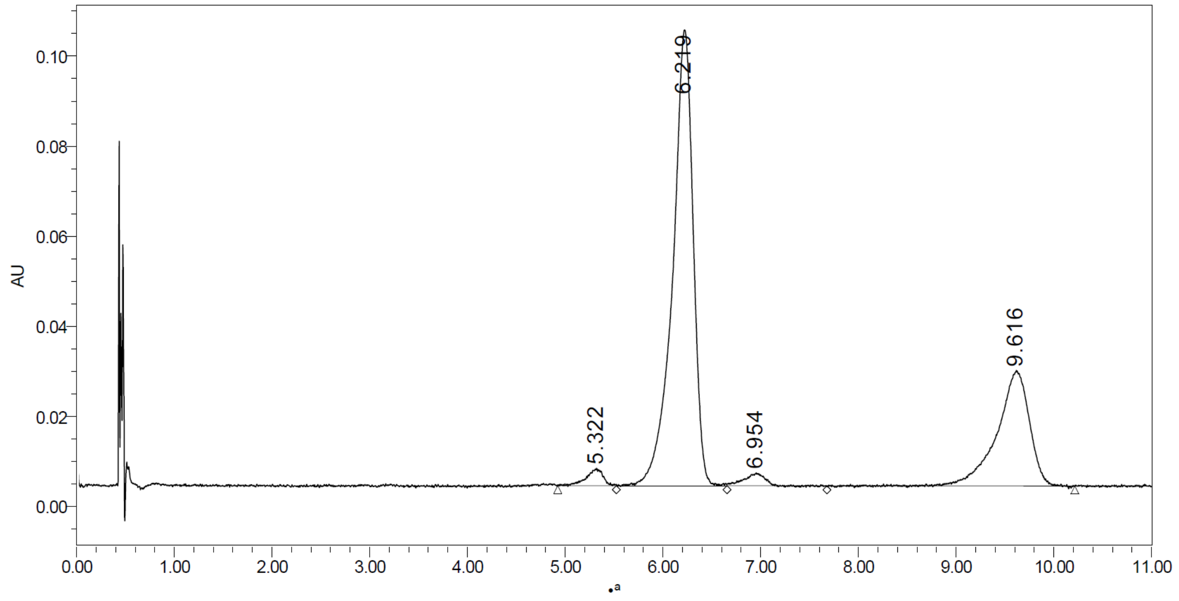

(S)-2-((R)-1-Naphthyl-2-nitroethyl)cyclohexanone (**3ad**)

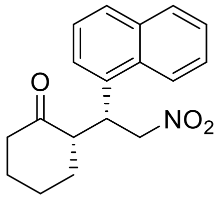

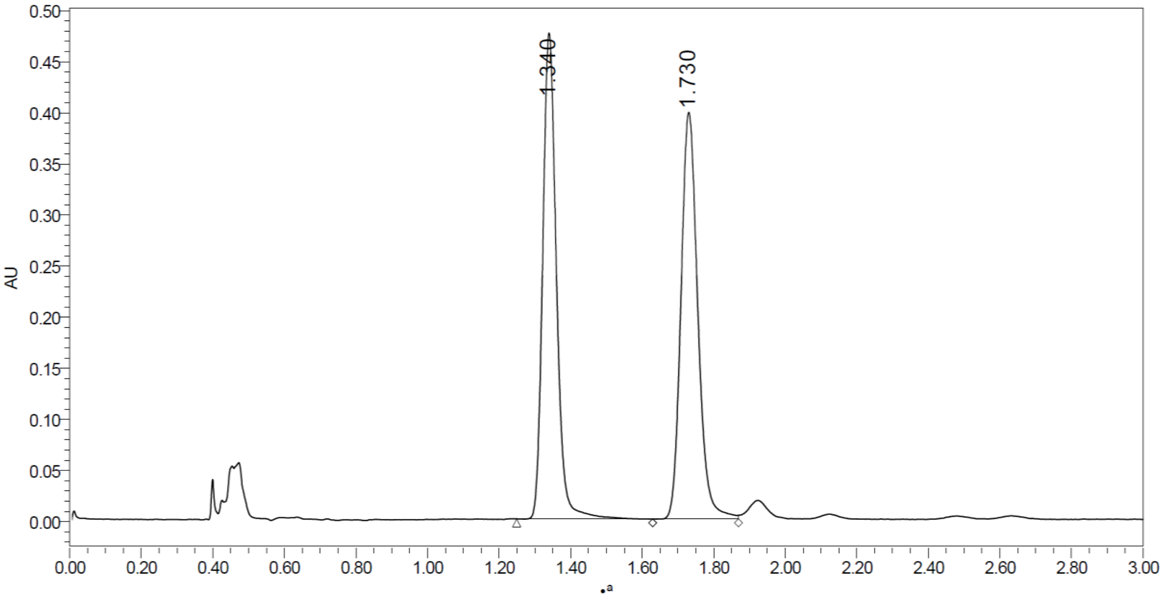

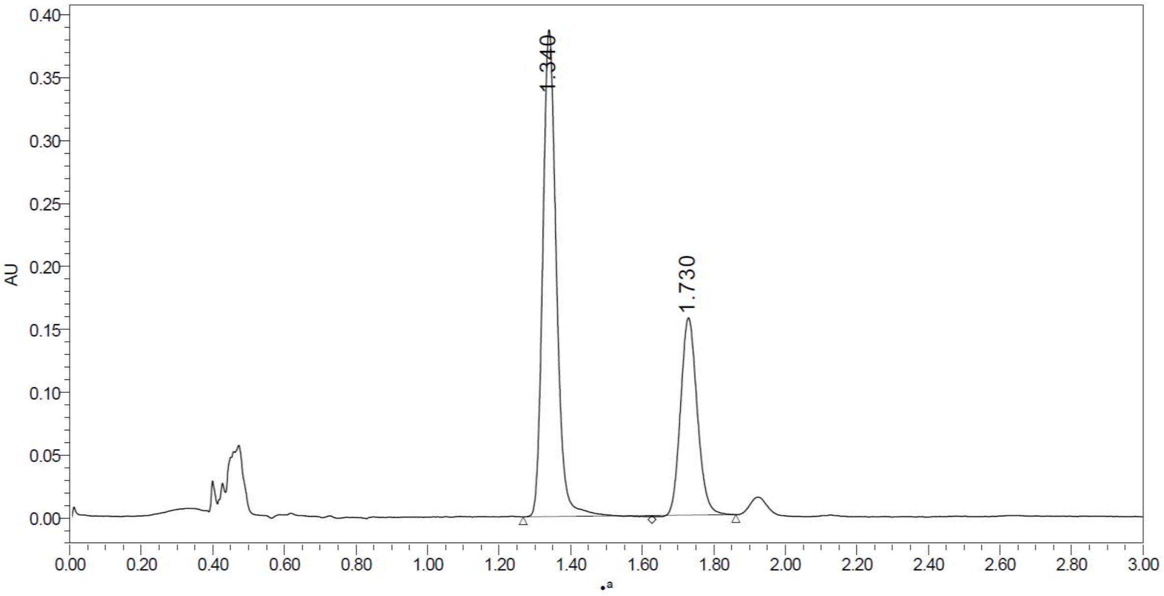

(S)-3-((R)-2-Nitro-1-phenylethyl)tetrahydro-4H-thiopyran-4-one (**3ba**)

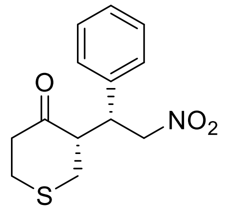

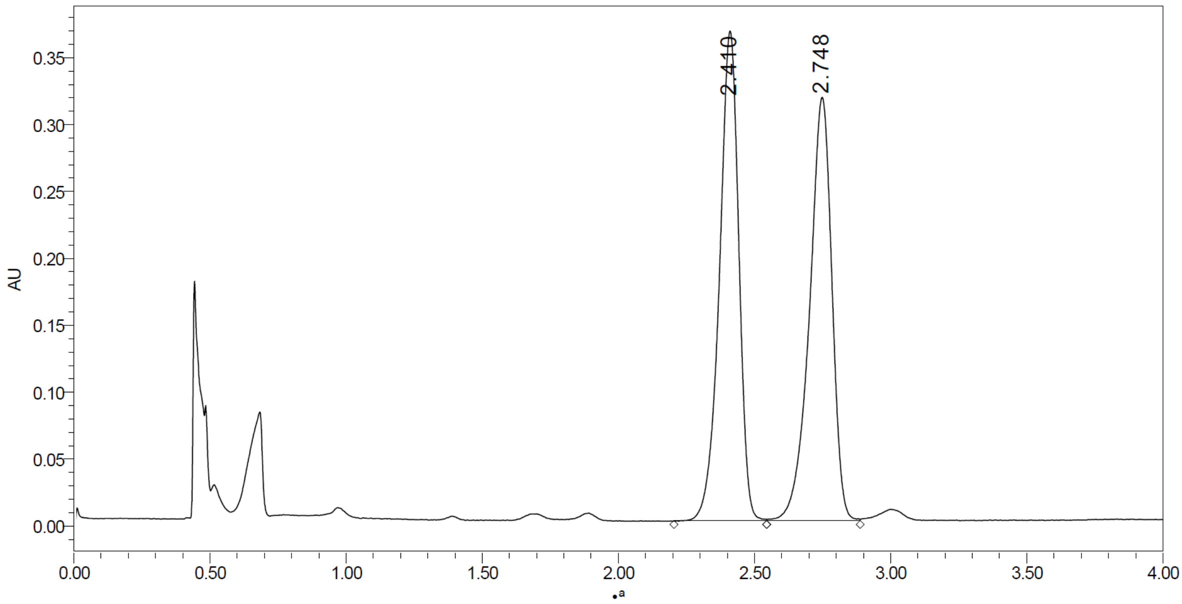

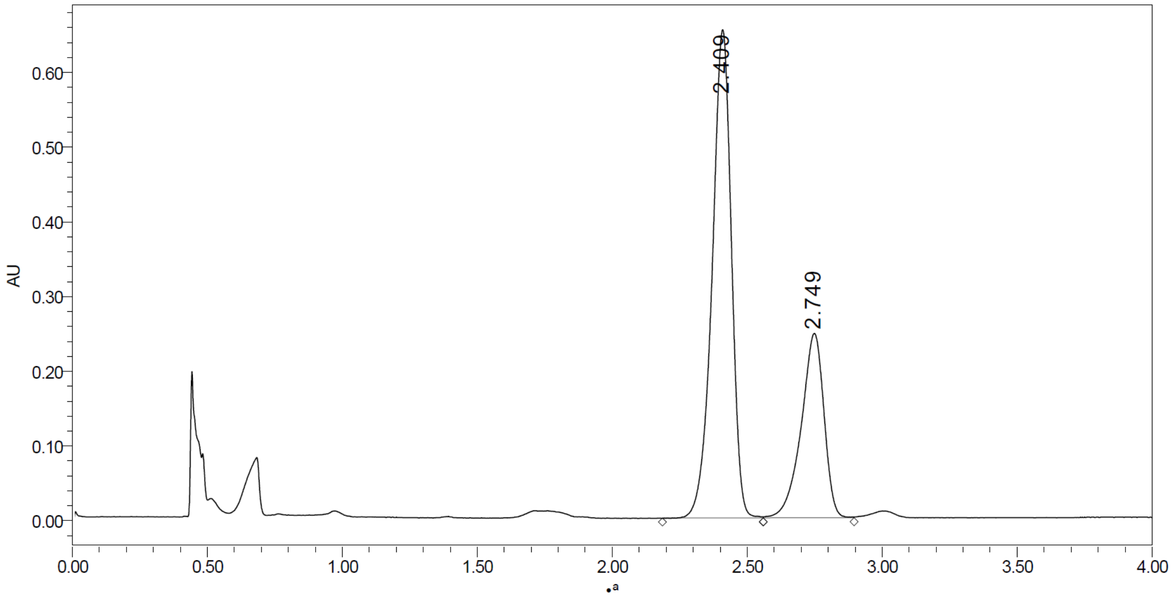

(S)-2-((R)-2-Nitro-1-phenylethyl)cyclopentan-1-one (**3ca**)

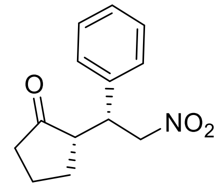

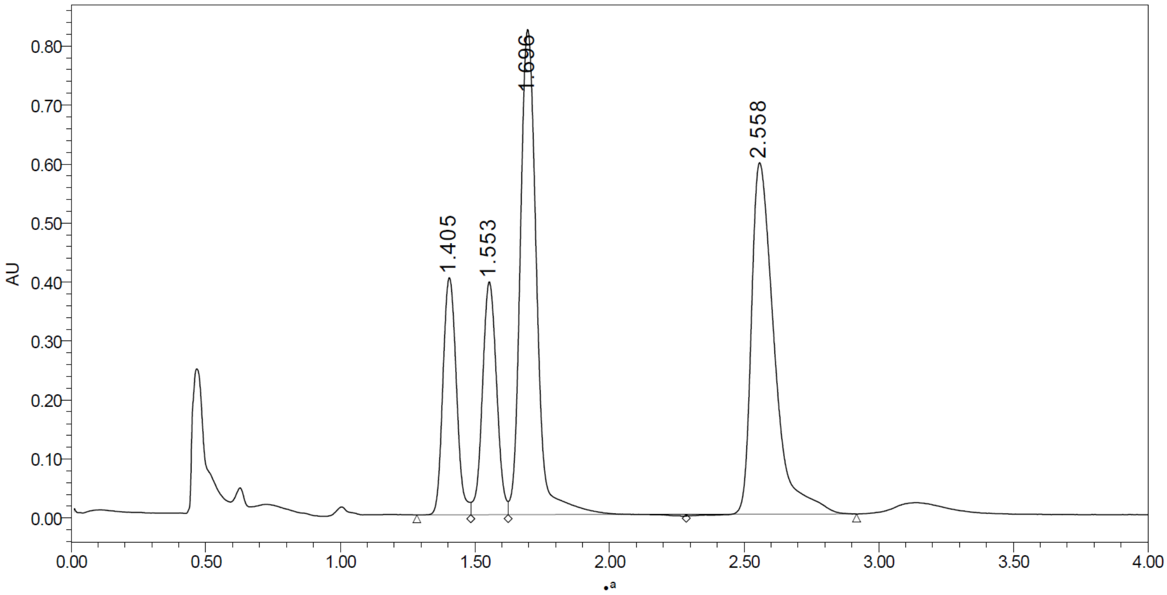

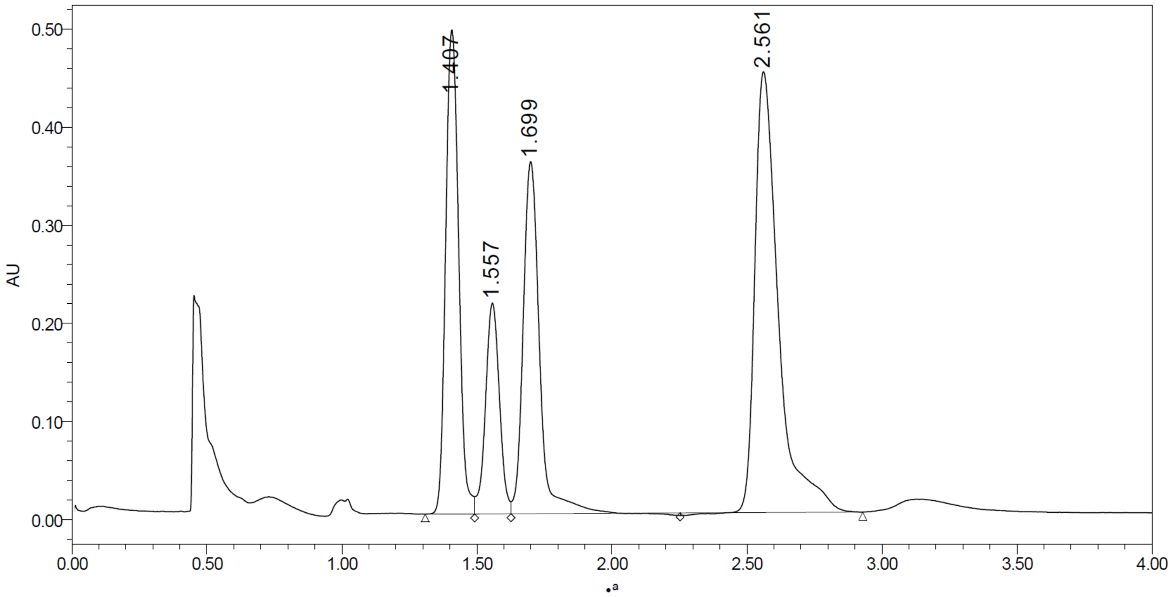

(R)-5-Nitro-4-phenylpentan-2-one (**3da**)

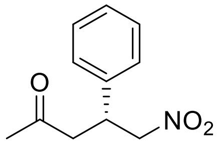

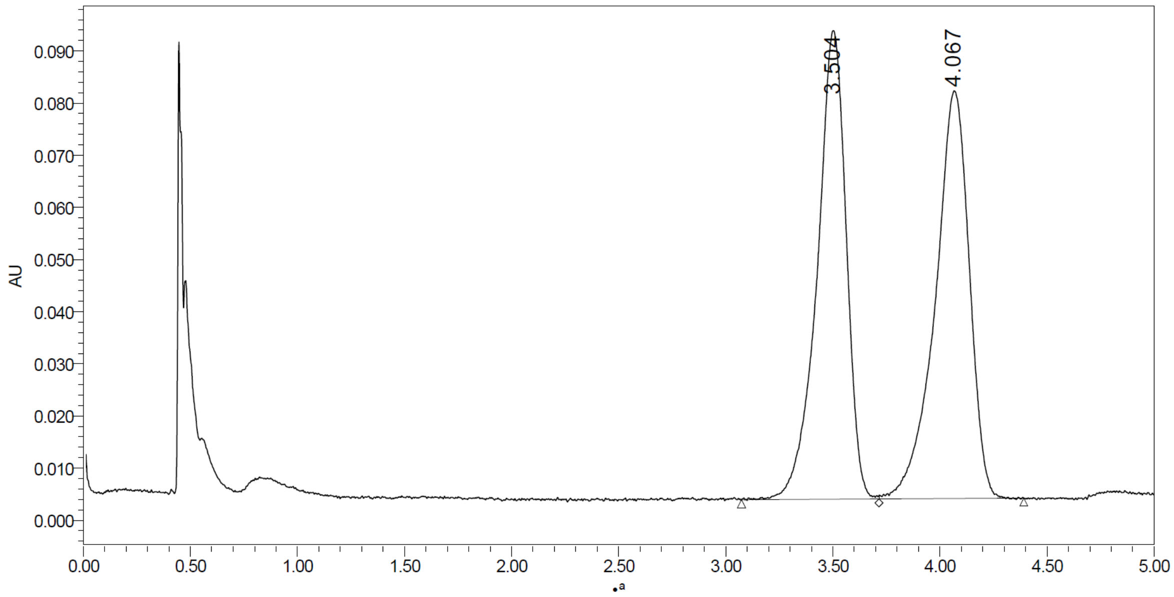

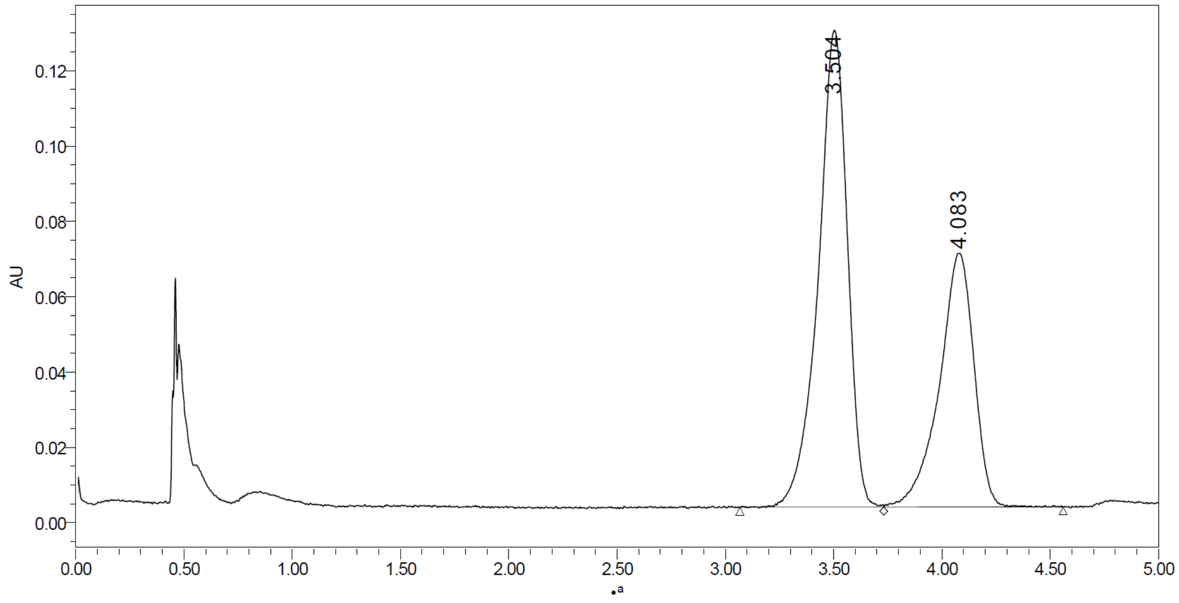

Appendix: List of reagents and chemicals

The following reagents were purchased from Sigma-Aldrich Co. LLC. (Tokyo, Japan);

ammonium chloride	SAJ first grade
4-bromobenzaldehyde	Reagentplus®
D-proline	Reagentplus®
sodium bromide	99% purity
sodium hypochlorite solution (available chlorine 10–15%)	reagent grade
tert-butanol	ACS reagent
2,2,6,6-tetramethylpiperidine 1-oxyl	98% purity

The following reagents were purchased from FUJIFILM Wako Pure Chemical Industries, Ltd. (Osaka, Japan);

acetone	guaranteed reagent
cyclohexanone	guaranteed reagent
dichloromethane	guaranteed reagent
N,*N*-dimethylformamide	guaranteed reagent
ethanol	guaranteed reagent
ethyl acetate	guaranteed reagent
hexane	guaranteed reagent
hydrochloric acid (35.0–37.0%)	guaranteed reagent
methanol	guaranteed reagent
methanol	for HPLC
trans-*p*-methoxy-β-nitrostyrene	95% purity
2-naphthaldehyde	97% purity
nitromethane	guaranteed reagent
trans-β-nitrostyrene	92% purity
piperidine	Wako special grade
DL-proline	Wako 1st grade
L-proline	Wako 1st grade
2-propanol	for HPLC
sodium hydroxide	guaranteed reagent
sodium sulfate	Wako 1st grade
toluene	guaranteed reagent

The following reagent was purchased from Merck KGaA (Darmstadt, Germany);

anhydrous Iron(III) chloride	98% purity

The following reagents were purchased from Tokyo Chemical Industry Co., Ltd. (Tokyo, Japan);

carboxymethycellulose sodium salt (n = ca. 500)	
cyclopentanone	guaranteed reagent
4-oxothiane	98% purity

References

1. Segal, L.; Creely, J.J.; Martin, A.E.; Conrad, C.M. An empirical method for estimating the degree of crystallinity of native cellulose using the X-ray diffractometer. *Text. Res. J.* **1959**, *29*, 786–794, doi:10.1177/004051755902901003.
2. Saito, T.; Isogai, A. TEMPO-mediated oxidation of native cellulose. The effect of oxidation conditions on chemical and crystal structures of the water-insoluble fractions. *Biomacromolecules* **2004**, *5*, 1983–1989, doi:10.1021/bm0497769.
3. Jalal, S.; Sarkar, S.; Bera, K.; Maiti, S.; Jana, U. Synthesis of nitroalkenes involving a cooperative catalytic action of iron(III) and piperidine: A one-pot synthetic strategy to 3-alkylindoles, 2*H*-chromenes and *N*-arylpyrrole. *Eur. J. Org. Chem.* **2013**, *2013*, 4823–4828, doi:10.1002/ejoc.201300172.
